# Optimization of Lutein Recovery from *Tetraselmis suecica* by Response Surface Methodology

**DOI:** 10.3390/biom11020182

**Published:** 2021-01-28

**Authors:** Kang Hyun Lee, Ye Won Jang, Hansol Kim, Jang-Seu Ki, Hah Young Yoo

**Affiliations:** Department of Biotechnology, Sangmyung University, 20, Hongjimun, 2-Gil, Jongno-Gu, Seoul 03016, Korea; oys7158@naver.com (K.H.L.); yesyewon@naver.com (Y.W.J.); biohansol0109@gmail.com (H.K.)

**Keywords:** antioxidant, lutein, optimization, response surface methodology, *Tetraselmis suecica*

## Abstract

Microalgae have been attracting attention as feedstock for biorefinery because they have various advantages, such as carbon fixation, high growth rate and high energy yield. The bioactive compounds and lutein contained in microalgae are known to be beneficial for human health, especially eye and brain health. In this study, in order to improve the recovery of bioactive extracts including lutein from *Tetraselmis suecica* with higher efficiency, an effective solvent was selected, and the extraction parameters such as temperature, time and solid loading were optimized by response surface methodology. The most effective solvent for lutein recovery was identified as 100% methanol, and the optimum condition was determined (42.4 °C, 4.0 h and 125 g/L biomass loading) by calculation of the multiple regression model. The maximum content of recovered lutein was found to be 2.79 mg/mL, and the ABTS radical scavenging activity (IC_50_) and ferric reducing antioxidant power (FRAP) value were about 3.36 mg/mL and 561.9 μmol/L, respectively. Finally, the maximum lutein recovery from *T. suecica* through statistical optimization was estimated to be 22.3 mg/g biomass, which was 3.1-fold improved compared to the control group.

## 1. Introduction

Biorefinery is defined as a biomass conversion process to obtain various products (e.g., chemicals, fuels and materials) from biomass [1]. Biorefinery has received a great deal of attention for its reusability, biodegradability, and reduction in greenhouse emissions [2]. However, the first-generation biomass used in biorefinery, such as corn, wheat, sugar cane and cassava, has caused competition for food source in countries with low income [3]. The utilization of second-generation biomass containing corn stover, rice husk, wheat straw and sugarcane bagasse cannot be economically produced on a large scale, because expensive technologies have been required, including pretreatment with enzymes or physicochemical treatments [4]. Microalgae, a third-generation biomass, are potential substrates for biorefinery due to advantages such as carbon fixation, high growth rates, low land use and high energy yield compared to terrestrial biomass [5,6]. In addition, various studies have been performed to convert microalgae into biofuels (bioethanol, biodiesel and biomethane) or valuable products (polysaccharide, biopolymers, antioxidant and pigment) due to its abnormal growth causing environmental pollution, involving disruption of aquatic ecosystem, mass mortality of fish and depletion of oxygen and poisonous water [6,7,8].

*Tetraselmis suecica*, the green algae, is widely used in aquaculture as a common nutrition source of aquatic species [9]. *T. suecica* consists of carbohydrates (22.4%), lipids (8.0%), proteins (48.7%), ash (17.5%) and bioactive compounds such as pigments (0.04%), vitamins and minerals [10]. Lutein, a fat-soluble pigment belonging to the carotenoid family, was known to have health benefits including antioxidant activity, anti-inflammatory properties and the prevention of cardiovascular and Alzheimer’s disease [11]. In 2017, the global carotenoid market value increased to approximately USD 1.5 billion, and lutein was estimated to account for about 15% of the total market [12]. Various studies have been reported to recover lutein from plants such as carrot, spinach and kale [13,14,15]. However, it is difficult to obtain lutein from plants because of existing small amounts in plants [16]. Microalgae such as *Chlorella fusca* (4.5 mg/g), *Tetracysis aplanosporum* (5.9 mg/g) and *Chlorococcum citroforme* (7.4 mg/g) have a higher lutein content than vegetables such as kale (0.03 mg/g), broccoli (0.04 mg/g) and cilantro (0.08 mg/g) and have a 5 to 10 times higher growth rate than plants; thus, various studies have been conducted to produce lutein from microalgae such as *Tetraselmis* sp., *Chlorella* sp. and *Muriellopsis* sp. [11,16,17]. However, since the cell wall of microalgae is very complex and its composition varies according to species, a standardized recovery method has not been suggested. In particular, the recovery of bioactive compounds contained in cells is not simple, and the loss rate and processing cost increase as the separation and purification process unit increase [17,18]. Therefore, improving the production of bioactive compounds such as lutein in cells is essential for industrial application, but it is also very important to improve the recovery efficiency from microalgal cells.

In general, extraction of bioactive compounds contained in bioresources such as carotenoids has been carried out in commercial applications by various methods, including organic solvents, ultrasonic waves, microwaves and supercritical fluids [18]. Among them, solvent extraction is a conventional technique used in the industry due to its low processing cost and simple operation [19]. However, since this extraction has disadvantages, such as the use of a large amount of solvent and long extraction time, the optimization process of variables to improve efficiency should be carried out for application to the industry [20]. Response surface methodology (RSM) is a multivariate statistical technique and is widely used in the optimization process to completely describe the effect of variables on the response and to reduce the number of experiments required [21,22].

In this study, *T. suecica*, which contains various carotenoids, was used as the feedstock for lutein recovery. Various solvents for liquid extraction, such as hexane, heptane, ethyl acetate (EA), acetone (Ace), ethanol (EtOH) and methanol (MeOH), were investigated to select the most effective solvent for lutein recovery. Statistical optimization of extraction conditions was performed to improve lutein recovery by RSM. In order to determine antioxidant activity of extracts, lutein contents, ABTS radical scavenging activity and FRAP value were investigated. Finally, the overall process of lutein recovery from biomass was evaluated with a material balance based on 1000 g *T. suecica* for industrial application.

## 2. Materials and Methods

### 2.1. Materials

*Tetraselmis suecica* was purchased from Chloland (Geoje-si, Gyeongsangnam-do, Korea). Hexane, heptane, EA, Ace, EtOH, MeOH, acetonitrile and sodium acetate were purchased from Samchun Chemical (Kangnam-gu, Seoul, Korea). Lutein, 2,2′-azino-bis (3-ethylbenzothiazoline-6-sulphonic acid) (ABTS), 2,4,6 tripyridyl-S-triazine (TPTZ), Iron(III) chloride (FeCl_3_) and Iron(II) sulfate (FeSO_4_) were purchased from Sigma-Aldrich (St. Louis, MO, USA). All reagents and chemicals in this study were used above analytical grade.

### 2.2. Liquid Recovery Procedure and Solvent Selection

Liquid extraction of lutein from *T. suecica* was performed with 20 mL screw-capped glass bottles in a water bath. Seven different solvents, distilled water (DW), hexane, heptane, EA, Ace, EtOH and MeOH, were used for the liquid recovery, and 1 g of *T. suecica* was soaked in 10 mL of each solvent at 4 °C for 1 h. The extract was centrifuged at 13,000 rpm for 5 min, and the supernatant was stored at −20 °C to analyze the lutein contents.

Effective solvents for lutein recovery were selected based on the lutein content, and to determine the optimum concentration of solvent, the selected solvents were diluted with DW and prepared in 10%, 50%, 90% and 100%, respectively. The extraction conditions and sample preparation for analysis were carried out in the same procedure as in the previous step. All experiments were performed in triplicate to indicate standard deviation. 

### 2.3. Experimental Design Using Response Surface Mothodology

The central composite design (CCD) of RSM was performed to optimize the lutein recovery using Design-Expert 7 software (Stat-Ease Inc., Minneapolis, MN, USA). The CCD creates statistical models of the interaction of independent factors on lutein extraction. The three factors and five different levels (−2, −1, 0, 1 and 2) to optimize the lutein extraction are shown in Table 1. The selected factors and their ranges are as follows: temperature (*X*_1_; 0–60 °C), time (*X*_2_; 1–5 h) and solid/liquid (S/L) ratio (*X*_3_; 50–150 g/L). The analysis of variance (ANOVA) was used to analyze the experimental results. Each factor and its interactions were expressed by applying the following quadratic equation:

*Y* = *β*_0_ + ∑ *β_i_X_i_* + ∑ *β*_ij_*X*_i_*X*_j_ + ∑ *β*_ii_*X*_i_^2^ (1)

where *Y* is the response factor (lutein contents or ABTS IC_50_), *X_i_* and *X_j_* are the coded levels of independent factor (temperature, time and S/L ratio), *β*_0_ is the interception coefficient, *β_i_* is the first order model coefficients, *β_ii_* is the quadratic coefficients for the factor *i* and *β_ij_* is the linear model coefficient for the interaction between factors *i* and *j* [23,24]. All experiments were performed in triplicate, and the average of lutein contents (mg/mL) and ABTS IC_50_ (mg/mL) were taken as the response, respectively. 

### 2.4. Analytical Methods

#### 2.4.1. Determination of Lutein Contents Using High Performance Liquid Chromatography

The lutein contents in the bioactive extracts were determined by a high performance liquid chromatography (HPLC) system equipped with a diode array detector (DAD, Hitachi, Tokyo, Japan) at 450 nm. An INNO Column C18 (5 μm, 4.6 mm × 250 mm, Young Jin Biochrom, Seongnam-si, Korea) was used to analyze lutein contents at 25 °C. A solvent program followed Schüler [16] methods, and the gradient elution was as follows: solvent A, acetonitrile: water (90:10, *v*/*v*) and solvent B, ethyl acetate; start at 100% A; 0–16 min, 0–60% B; 16–30 min, 60% B; 30–32 min, 100% B; 32–35 min, 100% A and 35–40 min, 100% A. The flow rate was maintained at 0.8 mL/min, and injection volume was 5 μL. The calibration curves for quantification were prepared using the lutein standard (Sigma-Aldrich, St. Louis, MO, USA).

#### 2.4.2. ABTS Radical Scavenging Activity

Antioxidant activity of the extract was measured by modifying the ABTS radical scavenging assay [25]. ABTS^•+^ cation solution was prepared by reacting 7 mM ABTS solution with 2.45 mM potassium persulfate (1:1) and stored at room temperature for 12 h before the reaction. ABTS^•+^ cation solution was diluted until optical density (OD) reached 0.8 at 734 nm. After adding 950 μL diluted ABTS^•+^ cation solution to a 50 μL sample, the mixture was reacted at 25 °C for 30 min and OD was measured at 734 nm using a spectrophotometer (DU 730, Beckman Coulter, Brea, CA, USA). The blank was 1 mL methanol, and the control was the mixture of 950 μL diluted ABTS^•+^ cation solution with 50 μL methanol. ABTS^•+^ radical scavenging activity was determined according to the following equation:

ABTS radical scavenging activity (%) = (1 − (Sample OD_734 nm_/Control OD_734 nm_)) × 100 (2)

ABTS^•+^ radical scavenging activity was converted to IC_50_ (mg/mL), which means the concentration of the sample neutralizing 50% free radical. 

#### 2.4.3. FRAP Assay

In order to investigate of antioxidant activity of lutein recovered under the optimum conditions, FRAP assay was performed [26]. FRAP solution was prepared by reacting 300 mM sodium acetate buffer (pH 3.6), 10 mM TPTZ solution in 40 mM HCl and 20 mM FeCl_3_ (10:1:1) and used within 3 h. An amount of 1 mL DW was added to a 5 mL tube and soaked at 37 °C for 5 min. Then, a 100 μL sample and 3 mL FRAP solution were added. The mixtures were reacted at 37 °C for 5 min and OD was measured at 593 nm. For the blank and quality control, DW and 1 mM FeSO_4_ solution were used instead of the sample, respectively. The FRAP value was determined according to the following equation:

FRAP value (μmol/L) = (Sample OD_593 nm_/Control OD_593 nm_) × Fe^2+^ standard concentration (μmol/L) (3)

## 3. Results and Discussion

### 3.1. Selection of Extract Solvent

**Figure 1:**
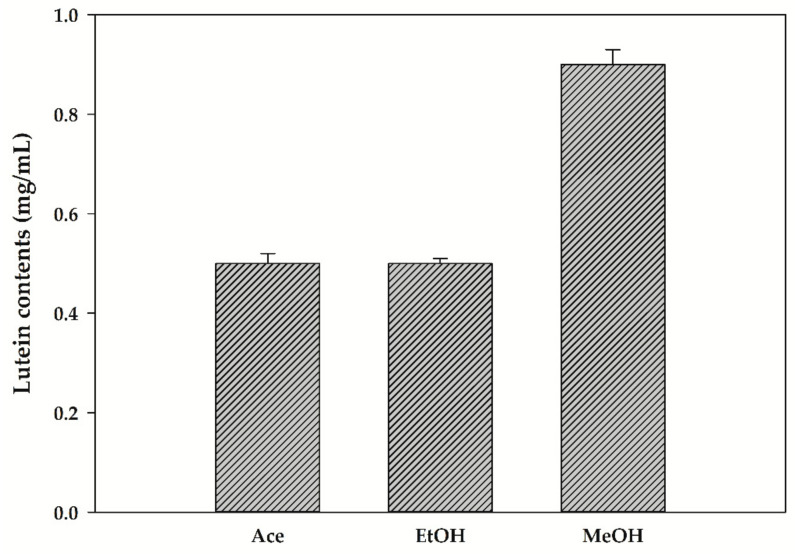
The effect of various solvents on lutein recovery from *Tetraselmis suecica*.

The contents of lutein recovered from *T. suecica* using various solvents are shown in Figure 1. Lutein was not recovered when DW, hexane, heptane and EA were used as solvents. The content of lutein recovered by Ace, EtOH and MeOH was found to be 0.5, 0.5 and 0.9 mg/mL, respectively. In carotenoid extraction, polar solvents such as Ace, EtOH and MeOH are more suitable for xanthophylls, including lutein and violaxanthin, whereas nonpolar solvents such as hexane, chloroform and tetrahydrofuran (THF) are more efficient for carotene and esterified carotenoids [26,27]. Xanthophyll is more polar than carotene due to an oxygen atom [28]. 

**Figure 2:**
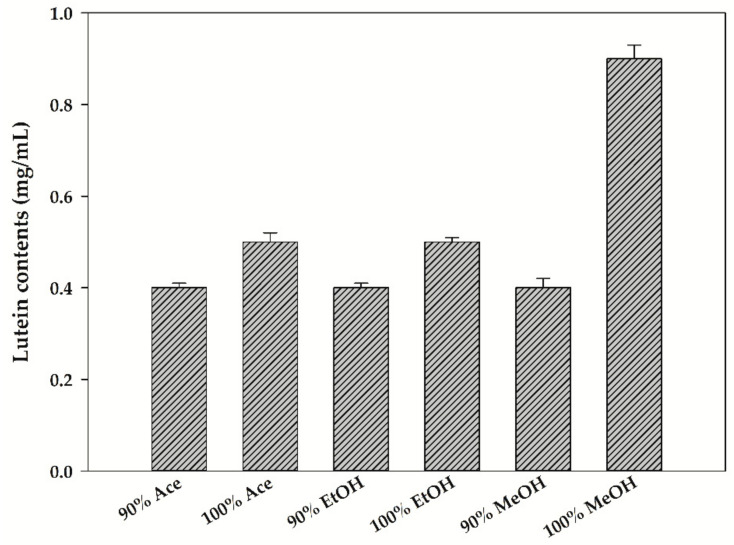
The effect of various concentrations of solvent on lutein recovery from *Tetraselmis suecica*.

Various studies have been reported using organic solvents and organic solvent–DW mixtures to effectively extract carotenoids from algal biomass [27]. To determine the most effective solvent and concentration for lutein recovery from *T. suecica*, three organic solvents (Ace, EtOH and MeOH) were used at various concentrations mixed with DW (Figure 2). Lutein was not recovered when an organic solvent containing 50% or more of DW was used. Lutein recovery was most effective when 100% organic solvent was used (not containing DW), and the contents of lutein recovered by 100% Ace, EtOH and MeOH were about 0.5, 0.5 and 0.9 mg/mL, respectively. Most of the carotenoids have a low water solubility because of their form of transisomer hydrocarbons in nature [29]. Therefore, the most effective solvent for lutein recovery from *T. suecica* was determined as 100% MeOH.

### 3.2. Determination of Lutein Recovery Conditions Using RSM

CCD of RSM was performed to optimize the recovery conditions of lutein from *T. suecica*. RSM has the advantage of reducing the number of experiments and obtaining reliable data [30]. Three factors (*X*_1_: temperature, *X*_2_: time and *X*_3_: S/L ratio) and five levels (temperature: 0, 15, 30, 45 and 60 °C; time: 1, 2, 3, 4 and 5 h; S/L ratio: 50, 75, 100, 125 and 150 g/L) were adopted for CCD. Table 2 shows the 20 designed experiments and their responses. 

The results of CCD were expressed as the following second-order polynomial equation by applying a quadratic regression ANOVA to the experimental data.

*Y_L_* = 1.67 − 0.012 *X*_1_ + 0.055 *X*_2_ + 0.38 *X*_3_ + 0.088 *X*_1_*X*_2_ + 0.026 *X*_1_*X*_3_ − 0.044 *X*_2_*X*_3_ – 0.11 *X*_1_^2^ − 0.061 *X*_2_^2^ − 0.039 *X*_3_^2^ (4)

*Y_A_* = 4.50 − 0.40 *X*_1_ − 0.082 *X*_2_ − 0.70 *X*_3_ − 0.25 *X*_1_*X*_2_ − 0.11 *X*_1_*X*_3_ − 0.069 *X*_2_*X*_3_ − 0.007333 *X*_1_^2^ + 0.005371 *X*_2_^2^ + 0.36 *X*_3_^2^ (5)

where *Y_L_* is the lutein contents (mg/mL), *Y_A_* is ABTS IC_50_ (mg/mL), which is the concentration of the extracts to show 50% inhibition of ABTS radical cations. *X*_1_, *X*_2_ and *X*_3_ are the independent factors and indicate temperature, time and S/L ratio, respectively. The results of ANOVA for response surface quadratic model are represented in Table 3 and Table 4.

The F-value represents that the model is significant, and the f-values of each model were 17.17 and 19.13, respectively. For the model terms to be significant, the *p*-value of model should be less than 0.05 [31]. In addition, a *p*-value higher than 0.1 means that the model terms are not significant, indicating that the individual lone factors are independent and have no effect [32,33]. The *p*-values of each model were both <0.0001, which shows that the model term is significant for both models. In lutein recovery, *X*_3_, *X*_1_^2^ and *X*_2_^2^ (*p* > 0.05) were significant model terms, and for ABTS radical scavenging activity, *X*_1_, *X*_3_, *X*_1_*X*_2_ and *X*_3_^2^ (*p* > 0.05) were significant model terms. The *p*-values of the lack-of-fit of each model were 0.2923 and 0.0764, respectively, which were not significant (*p*-value > 0.05) relative to the pure error. This means that the quadratic model is statistically significant for the response [34]. The coefficients of variation (CV) for each model were determined as 9.08% and 6.11%, which was less than 10%, meaning that the variation of the experimental data is within a rational range [35]. The coefficient of determination (R^2^) refers to the amount of variance that the model describes, and adjusted R^2^ is modified by the degree of freedom [36]. R^2^ should be higher than 0.9, indicating that the model has high reliability, and the difference between R^2^ and adjusted R^2^ should be less than 0.2 [37]. In each model, R^2^ was 0.9392 and 0.9451, and adjusted R^2^ was 0.8845 and 0.8957, respectively, and the difference between R^2^ and adjusted R^2^ for each model did not exceed 0.2. Each model had adequate precision of 16.004 and 19.864, respectively. Adequate precision determines the signal-to-noise ratio, and a ratio greater than four means that the predictive model is suitable for exploring the designed space [38].

**Figure 3:**
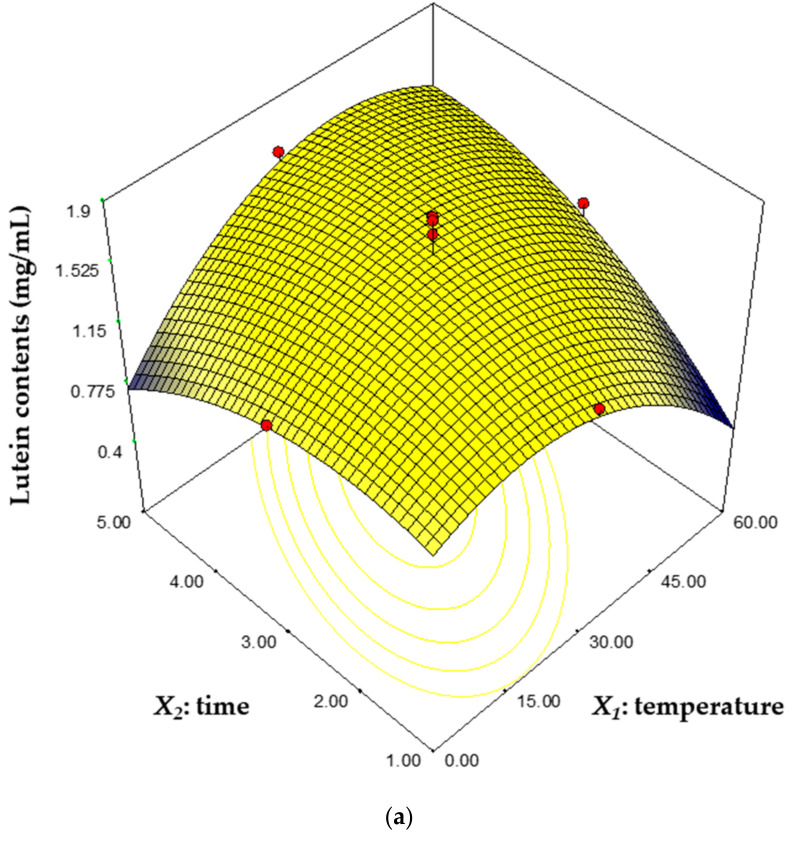

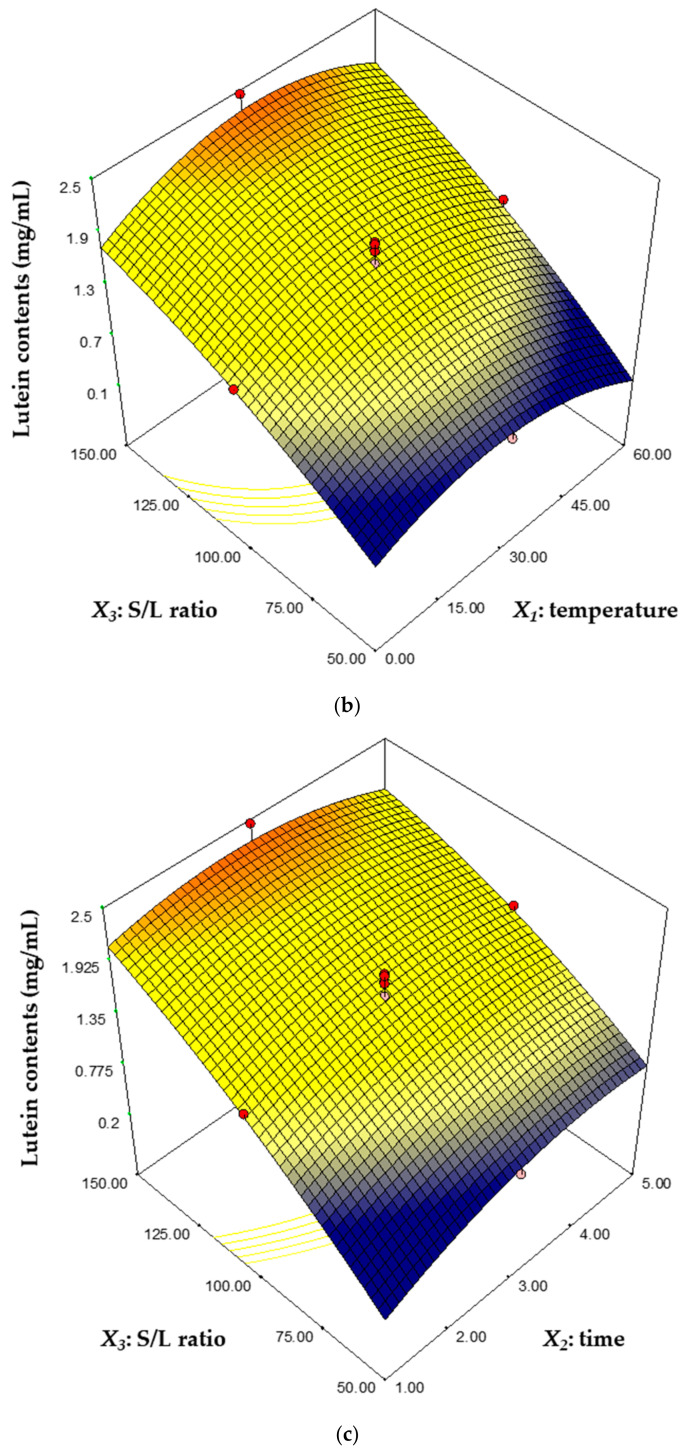
The three-dimensional response surface plots representing the effects of each factor on the lutein contents. Effects of temperature and time (**a**), temperature and S/L ratio (**b**) and time and S/L ratio (**c**).

**Figure 4:**
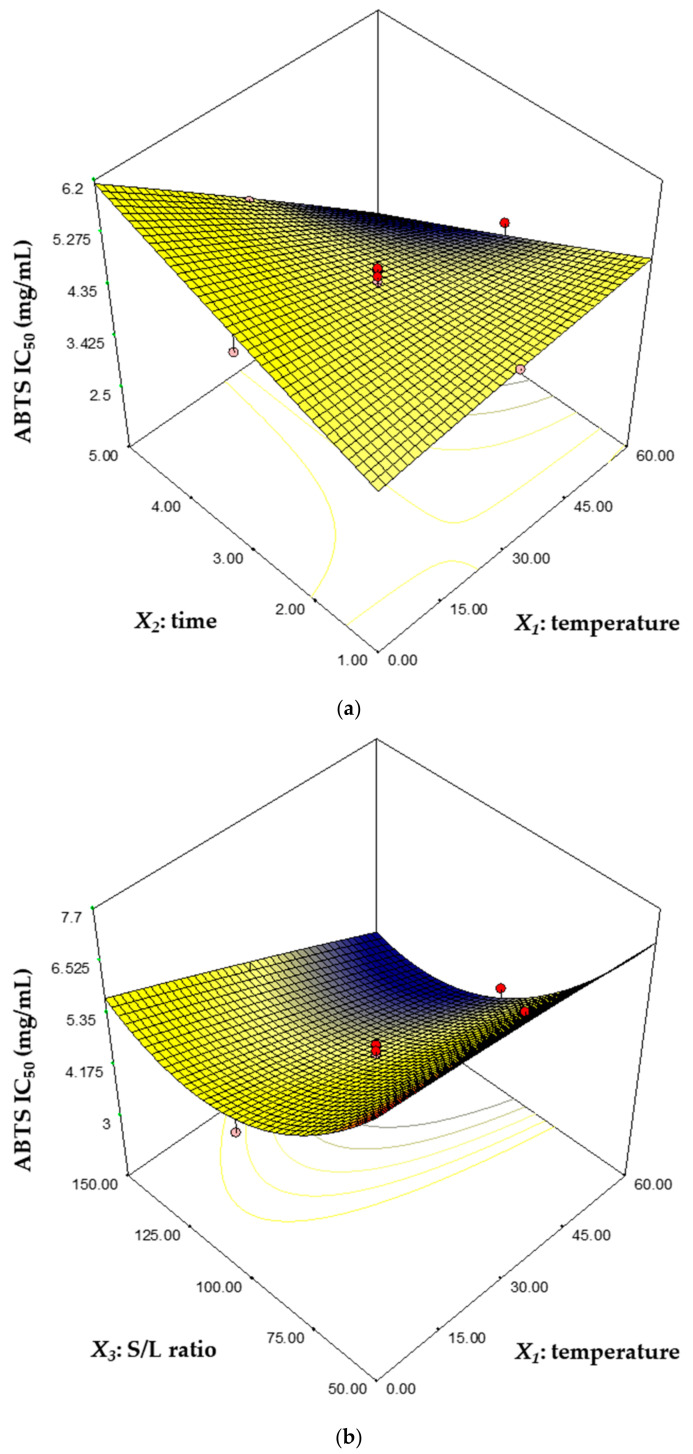

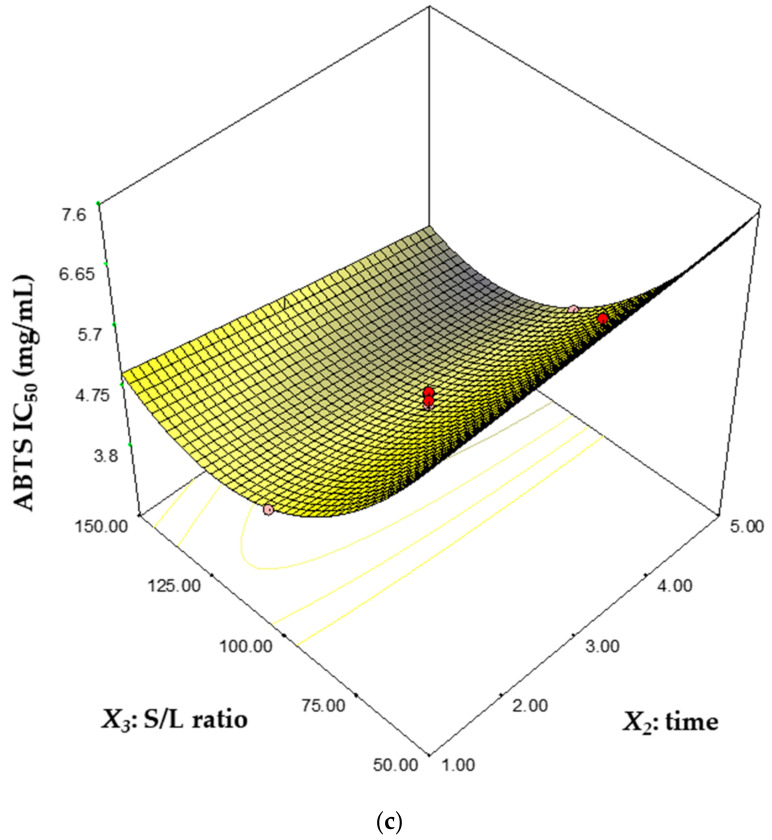
The three-dimensional response surface plots representing the effects of each factor on ABTS radical scavenging activity. Effects of temperature and time (**a**), temperature and S/L ratio (**b**) and time and S/L ratio (**c**).

The effect of interactions between factors on the response was plotted as a three-dimensional plot based on an established regression model (Figure 3 and Figure 4). The three-dimensional plot is suitable for surface response analysis studies because it shows the possible independence of the factor with the response [39]. In Figure 3a, the lutein contents were highest at 30 °C for 3 h and decreased with the change of the temperature and time, and especially, it decreased rapidly at 60 °C for 1 h and at 0 °C for 5 h. Figure 3b shows that the lutein contents increased as the temperature approached 30 °C and as the solid–liquid ratio increased. The effects of a variation in the time and S/L ratio on the lutein contents is represented in Figure 3c. The lutein contents increased as the time reached 3 h, and as the S/L ratio increased. Figure 4a shows the effect of temperature and time on ABTS radical scavenging activity. ABTS radical scavenging activity was lowest at 5 h and 60 g/L. It is estimated that the exposure of lutein to heat for a long time reduced the antioxidant activity due to thermal degradation of lutein [11]. The low level of temperature and S/L ratio increased ABTS radical scavenging activity, while the high level of temperature and S/L ratio drastically reduced ABTS radical scavenging activity (Figure 4b). In Figure 4c, the ABTS radical scavenging activity was lowest when the S/L ratio was 125 g/L, whereas it increased rapidly as the S/L ratio decreased, and the time did not have a significant effect on the ABTS radical scavenging activity.

The numerical optimization was performed by using established model equations to maximize lutein contents and ABTS radical scavenging activity at the designed level of the factors (Table 5). The optimum conditions are as follows: temperature of 42.4 °C, time of 4.0 h and S/L ratio of 125.0 g/L. Under the optimum conditions, the maximum recovered lutein content and ABTS radical scavenging activity were estimated as 1.97 and 3.39 mg/mL, respectively. The three independent replicates were performed under the optimum conditions to verify the reliability of the predicted model. The average values of lutein contents and ABTS radical scavenging activity were 2.79 and 3.36 mg/mL, respectively. These results show that the model equation established in this study is applicable to lutein recovery from *T. suecica*.

In order to analyze the antioxidant activity contained in the bioactive extracts under optimum conditions, various biological analyses were performed by using ABTS and FRAP methods (Table 6). The lutein standard was prepared at the same concentration (2.79 mg/mL) as the lutein recovered from *T. suecica* to compare the antioxidant activity. ABTS radical scavenging activity and FRAP value of the lutein standard were 3.44 mg/mL and 320.5 μmol/L, respectively. ABTS radical scavenging activity and FRAP value of the lutein recovered from *T. suecica* were 3.36 mg/mL and 561.9 μmol/L, respectively. The bioactive extracts from *T. suecica* showed about 1.8-fold higher antioxidant activity than the lutein standard in the FRAP assay. The extract fraction contained lutein as well as other bioactive compounds; thus, it is believed to have higher antioxidant activity. In order to utilize these extracts industrially, such as functional foods, cosmetics and pharmaceuticals, first, the purity of lutein should be determined according to the purpose, and then purified through an appropriate downstream process.

### 3.3. Evaluation of the Overall Process and Material Balance

**Figure 5:**
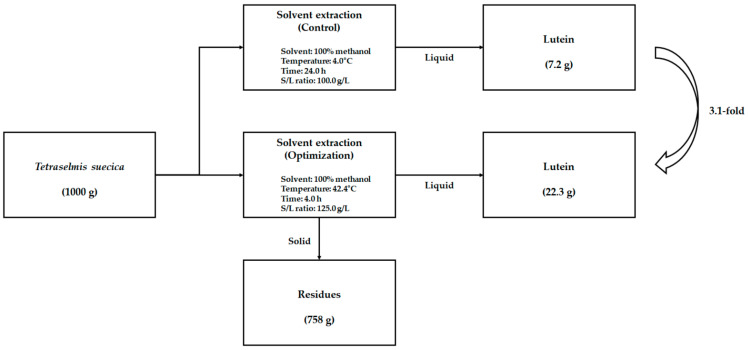
Material balance of microalgae to lutein based on 1000 g *Tetraselmis suecica*.

Figure 5 shows the overall process for lutein recovery from *T. suecica* evaluated by material balance. In the control group (temperature: 4 °C, time: 24 h and S/L ratio: 100 g/L), lutein recovery was estimated to be about 7.2 g based on 1000 g of biomass. Under statistically optimized extraction (temperature: 42.4 °C, time: 4.0 h and S/L ratio: 125.0 g/L), the lutein recovery was estimated to be about 22.3 g based on 1000 g of biomass, which showed a 3.1-fold increase compared to the control group. After solvent extraction, 758 g of residue was generated, which still contained compounds such as carbohydrates, lipids and proteins [40]. We are planning a follow-up study to develop a biorefinery platform that uses these residues as raw materials and converts them into valuable products.

Microalgae produce carotenoids including lutein, zeaxanthin and astaxanthin for chlorophyll protection, phototropism and phototaxis [41]. Lutein has been recovered from microalgae such as *Auxenochlorella* sp., *Chlorella* sp., *Desmodesmus* sp. and *Tetraselmis* sp., and related studies are summarized in Table 7. Dried, freeze-dried and wet biomass types were used in the lutein recovery process. The main parameters of the extraction were solvent, temperature, time and S/L ratio. Ace, MeOH, diethyl ether and THF were used as extraction solvents. The previous study focused on changes in lutein recovery due to culture conditions or environmental stress, so lutein recovery was only 0.60–7.50 mg/g biomass. In contrast, our study focused on the mass recovery of lutein and performed the lutein extraction under a wide range of extract conditions (temperature: 0–60 °C, time: 1–4 h and S/L ratio: 50–150 g/L) (Figure 3 and Figure 4). The maximum lutein recovery was determined to be 22.3 mg/g biomass under the optimum conditions (temperature: 42.4 °C, time: 4.0 h and S/L ratio: 125.0 g/L), a 3.1-fold increase compared to the control. These results show that our study is suitable for mass recovery of lutein from microalgae for industrial application.

## 4. Conclusions

In this study, we suggested a statistical model that predicts the optimum reaction conditions for lutein extraction from *Tetraselmis suecica*. The content of lutein extracted from *T. suecica* using various solvents, such as acetone, ethanol, and methanol, was found to be 0.5, 0.5 and 0.9 mg/mL, respectively, and the most effective solvent was determined as 100% methanol. The central composite design of response surface methodology was performed to improve the lutein extraction. The effect of the extraction parameters, including temperature, time and S/L ratio, on the lutein contents and ABTS radical scavenging activity was investigated. The optimum condition for lutein extraction from *T. suecica* was determined as the temperature of 42.4 °C, time of 4.0 h and S/L ratio of 125.0 g/L. Under optimum conditions, the maximum extraction of lutein was achieved at about 2.97 mg/mL, which was about 3.1-fold higher than the control group (before optimization), and the ABTS radical scavenging activity (IC_50_) was found to be 3.36 mg/mL. This study is expected to be usefully applied to the development of eco-friendly biorefinery by enhancing the recovery efficiency of lutein, a high value-added material from microalgae through statistical methods. The goal of further study in near future is to investigate the applicability of predictive models to other microalgae with similar cellular structures and to develop the extracted lutein, as well as the residue from microalgae, as a source for various bioapplications.

## Figures and Tables

**Table 1:** Factors and their levels in the response surface methodology (RSM).

Factors	Unit	Symbol	Coded Factor Levels				
			−2	−1	0	1	2
Temperature	°C	*X*_1_	0	15	30	45	60
Time	h	*X*_2_	1	2	3	4	5
S/L ratio	g/L	*X*_3_	50	75	100	125	150

**Table 2:** Experimental designs and their responses for five-level, three-factor response surface analysis.

Std	Coded Factor Levels			Response		
	*X*_1_	*X*_2_	*X*_3_	Lutein Contents (mg/mL)	ABTS IC_50_ (mg/mL)	
1	−1	−1	−1	1.08	5.56	
2	1	−1	−1	0.90	5.40	
3	−1	1	−1	1.20	6.22	
4	1	1	−1	1.17	4.71	
5	−1	−1	1	1.86	4.97	
6	1	−1	1	1.58	4.02	
7	−1	1	1	1.61	5.02	
8	1	1	1	1.87	3.39	
9	−2	0	0	1.25	4.96	
10	2	0	0	1.27	3.90	
11	0	−2	0	1.35	4.66	
12	0	2	0	1.58	4.30	
13	0	0	−2	0.66	7.60	
14	0	0	2	2.45	4.23	
15	0	0	0	1.51	4.65	
16	0	0	0	1.56	4.70	
17	0	0	0	1.81	4.46	
18	0	0	0	1.70	4.35	
19	0	0	0	1.71	4.55	
20	0	0	0	1.78		4.22

**Table 3:** ANOVA for response surface quadratic model of the lutein contents.

Source	Sum of Squares	Degree of Freedom	Mean Squares	F-Value	*p*-Value	Remarks
Model	2.85	9	0.32	17.17	<0.0001	significant
*X*_1_	0.002238	1	0.002238	0.13	0.7293	
*X*_2_	0.048	1	0.048	2.62	0.1365	
*X*_3_	2.36	1	2.36	128.06	<0.0001	significant
*X*_1_ *X*_2_	0.062	1	0.062	3.34	0.0976	
*X*_1_ *X*_3_	0.005529	1	0.005529	0.30	0.5962	
*X*_2_ *X*_3_	0.016	1	0.016	0.85	0.3774	
*X*_1_^2^	0.32	1	0.32	17.18	0.0020	significant
*X*_2_^2^	0.094	1	0.094	5.08	0.0479	significant
*X*_3_^2^	0.038	1	0.038	2.04	0.1840	
Residual	0.18	10	0.018			
Lack of fit	0.12	5	0.023	1.68	0.2923	not significant
Pure error	0.069	5	0.014			
Total	3.04	19				

Coefficients of variation (CV): 9.08%. Coefficient of determination (R^2^): 0.9392. Adjusted R^2^: 0.8845. Adequate precision: 16.004.

**Table 4:** ANOVA for response surface quadratic model of ABTS radical scavenging activity.

Source	Sum of Squares	Degree of Freedom	Mean Squares	F-Value	*p*-Value	Remarks
Model	14.75	9	1.64	19.13	<0.0001	significant
*X*_1_	2.52	1	2.52	29.46	0.0003	significant
*X*_2_	0.11	1	0.11	1.27	0.2863	
*X*_3_	7.85	1	7.85	91.67	<0.0001	significant
*X*_1_ *X*_2_	0.51	1	0.51	6.00	0.0343	significant
*X*_1_ *X*_3_	0.10	1	0.10	1.21	0.2980	
*X*_2_ *X*_3_	0.039	1	0.039	0.45	0.5178	
*X*_1_^2^	0.001325	1	0.001325	0.016	0.9025	
*X*_2_^2^	0.0007254	1	0.0007254	0.008467	0.9285	
*X*_3_^2^	3.33	1	3.33	38.83	<0.0001	significant
Residual	0.86	10	0.086			
Lack of fit	0.69	5	0.14	4.02	0.0764	not significant
Pure error	0.17	5	0.034			
Total	15.61	19				

Coefficients of variation (CV): 6.11%. Coefficient of determination (R^2^): 0.9451. Adjusted R^2^: 0.8957. Adequate precision: 19.864.

**Table 5:** Numerical optimization of lutein extraction based on multiple regression models.

**Parameters**	**Coded Factor Levels**	**Actual Factor Levels**
Temperature	0.8	42.4 °C
Time	1.0	4.0 h
S/L ratio	1.0	125.0 g/L
**Response**	**Predicted**	**Experimental**
Lutein contents (mg/mL)	1.97	2.79
ABTS IC_50_ (mg/mL)	3.39	3.36

**Table 6:** Antioxidant activity of the lutein standard and lutein extract.

	Lutein Standard	Lutein Extract
ABTS IC_50_ (mg/mL)	3.44	3.36
FRAP value (μmol/L)	320.5	561.9

**Table 7:** Summary of lutein extraction from microalgae.

Feedstock	Biomass Type	Extraction Method	Lutein Contents (mg/g biomass)	Ref.
*Auxenochlorella protothecoides*	dried	Solvent: Ace:MeOH (8:2, *v*/*v*)	3.32	[42]
*Chlorella sorokiniana* MB-1	freeze-dried	Solvent: THF, Temperature: 25 °C, Time: 0.67 h, and S/L ratio: 1 g/L	5.21	[43]
*Chlorella salina*	-	Solvent: MeOH:10 M KOH (2:1, *v*/*v*), Temperature: 40 °C, Time: 0.5 h, and S/L ratio: 66.7 g/L	2.92	[44]
*Chlorella zofingiensis* B32	freeze-dried	Solvent: Ethyl ether:MeOH (8:3, *v*/*v*)	4.38	[45]
*Desmodesmus* sp. F51	freeze-dried	Solvent: Diethyl ether, and S/L ratio: 5 g/L	7.50	[46]
*Tetraselmis* sp. CTP4	wet	Solvent: THF, and S/L ratio: 1 g/L	0.60	[27]
*Tetraselmis* sp. CTP4	wet	Solvent: Ace, and S/L ratio: 0.67 g/L	3.17	[16]
*Tetraselmis* sp. DS3	-	Solvent: 80% Ace	4.80	[47]
*Tetraselmis suecica* CS-187	freeze-dried	Solvent: Ace, S/L raio: 10 g/L	3.81	[48]
*Tetraselmis suecica*	freeze-dried	Solvent: MeOH, Temperature: 42.4 °C, Time: 4 h, and S/L ratio: 125 g/L	22.3	This study

## Data Availability

The data presented in this study are available on request from the corresponding author.
